# Central Regulation of PCOS: Abnormal Neuronal-Reproductive-Metabolic Circuits in PCOS Pathophysiology

**DOI:** 10.3389/fendo.2021.667422

**Published:** 2021-05-28

**Authors:** Baoying Liao, Jie Qiao, Yanli Pang

**Affiliations:** ^1^ Center for Reproductive Medicine, Department of Obstetrics and Gynecology, Peking University Third Hospital, Beijing, China; ^2^ National Clinical Research Center for Obstetrics and Gynecology, Peking University Third Hospital, Beijing, China; ^3^ Key Laboratory of Assisted Reproduction (Peking University), Ministry of Education, Beijing, China; ^4^ Beijing Key Laboratory of Reproductive Endocrinology and Assisted Reproductive Technology, Peking University Third Hospital, Beijing, China

**Keywords:** polycystic ovary syndrome, hypothalamus–pituitary–ovary axis, ovarian dysfunction, metabolic disorders, gut microbiota

## Abstract

Polycystic ovary syndrome (PCOS) is a common reproductive endocrine disease. PCOS patients are characterized by hyperandrogenemia, anovulation, and metabolic dysfunction. Hypothalamus–pituitary–ovary axis imbalance is considered as an important pathophysiology underlying PCOS, indicating that central modulation, especially the abnormal activation of hypothalamic GnRH neurons plays a vital role in PCOS development. Increased GnRH pulse frequency can promote LH secretion, leading to ovarian dysfunction and abnormal sex steroids synthesis. By contrast, peripheral sex steroids can modulate the action of GnRH neurons through a feedback effect, which is impaired in PCOS, thus forming a vicious cycle. Additionally, hypothalamic GnRH neurons not only serve as the final output pathway of central control of reproductive axis, but also as the central connection point where reproductive function and metabolic state inter-regulate with each other. Metabolic factors, such as insulin resistance and obesity in PCOS patients can regulate GnRH neurons activity, and ultimately regulate reproductive function. Besides, gut hormones act on both brain and peripheral organs to modify metabolic state. Gut microbiota disturbance is also related to many metabolic diseases and has been reported to play an essential part in PCOS development. This review concludes with the mechanism of central modulation and the interaction between neuroendocrine factors and reproductive or metabolic disorders in PCOS development. Furthermore, the role of the gut microenvironment as an important part involved in the abnormal neuronal–reproductive–metabolic circuits that contribute to PCOS is discussed, thus offering possible central and peripheral therapeutic targets for PCOS patients.

## Introduction

Polycystic ovary syndrome (PCOS) is a common reproductive and endocrine disorder, affecting 6–10% women of reproductive age worldwide (1). It is often characterized by menstrual disorder and infertility, abnormal elevated androgen levels, as well as polycystic ovary morphology (2). Metabolic disorders including IR, obesity, and abnormal lipid metabolism are represented in a considerably large part of PCOS patients. Besides, the long-term risk of type 2 diabetes, cardiovascular diseases, obstetrical complications, and endometrial cancer is significantly increased in women with PCOS than in control (2). The present therapy of PCOS mainly focuses on management of symptoms and prevention of long-term complications, including lifestyle modification, ovulation induction, anti-androgen therapy, and treatment of metabolic disorders (3); etiological treatment is still lacking.

Although it has been a long time since PCOS was discovered, the pathophysiology of PCOS remains unclear. Familial clustering and twin studies indicate the pivotal role genetic factor played in the etiology of PCOS; GWAS also identified PCOS’ candidate loci, which provide the studying bases for mechanism research (4, 5). Follicle growth is a complicated process which needs the coordination of LH and FSH, androgen, estrogen, AMH, and other possible factors; follicle growth is impaired in PCOS, leading to follicular arrest, ovulatory dysfunction, and PCOM (6). Induced by inhibition of aromatase activity, hyperandrogenemia and hyperinsulinemia usually impact and facilitate each other and then promote PCOS development (7). Rising pieces of evidence suggest the correlation between gut microbiota and PCOS (8, 9); our studies demonstrated that the gut microbiota–bile acid–IL-22 axis is involved in PCOS development *via* the crosstalk of gut innate immune system and ovary function (9, 10), providing strong evidence for the contribution of the gut microbiota in PCOS pathogenesis, while other possible pathways involved in gut microbiota need to be further explored.

In addition, HPO axis imbalance is considered as an important pathophysiology underlying PCOS. Hypothalamic GnRH neurons act as a central regulator of LH synthesis because of abnormally increased GnRH pulse, LH pulse frequency, and amplitude in women with PCOS, which further enhance androgen synthesis in ovarian theca cell and promote hyperandrogenemia, ovarian dysfunction, and metabolic disorders in women with PCOS (3, 11). GnRH neurons also mediate the effect of peripheral signals on CNS in PCOS development. Here we summarize the mechanism that abnormal neuronal–reproductive–metabolic circuits contributes to PCOS pathogenesis and shed light on central regulation of GnRH neurons mediated by gut microenvironment *via* the gut–brain axis, thus providing new insights into PCOS pathogenesis and treatment.

## The Effect of GnRH in PCOS Pathophysiology

A mounting body of evidence supports that increased GnRH pulse frequency and amplitude can promote LH synthesis over FSH synthesis, leading to a high LH/FSH ratio in women with PCOS (12). Elevating LH levels plays a vital role in the development of reproductive and metabolic disorders, based on the evidence listed below. First, LH promotes the synthesis of androgen in ovarian theca cells, which leads to hyperandrogenemia and arrested follicle development (11). Second, increased LH pulse frequency impairs estrogen and FSH synthesis, thus inhibiting follicle growth and ovulation. Third, LH promotes ovarian secretion of IGF-1 which can further promote LH binding and androgen synthesis in theca cell, and finally contributes to the formation of polycystic ovaries in PCOS patients (13). However, it’s still unclear whether the abnormal GnRH function is primary dysfunction of hypothalamus and pituitary or secondary to the complicated effect of reproductive and metabolic disorder, as well as unbalanced immune system and intestinal microenvironment in PCOS patients.

### Neuropeptide

#### Kisspeptin

Located in the hypothalamus, GnRH neurons serve as the final output pathway of central control of the reproductive axis and play a vital role in the control of puberty onset and gonadal function (14). Kisspeptin is the key upstream regulator in GnRH pulse formation: kisspeptin acts through G-protein-coupled receptors GRP54, also known as Kiss1R, to activate hypothalamic GnRH secretion (15); besides, they also transmit peripheral steroid hormone information to the hypothalamus and mediate the steroid feedback control of GnRH secretion (16, 17). Kisspeptin neurons are primarily located in the ARC and the AVPV/PeN of the hypothalamus, while these two clusters of kisspeptin neurons have different effects on the activation of GnRH neurons. Co-expressed with NKB and dynorphin, kisspeptin neurons in the ARC are usually described as one member of the KNDy system that regulates GnRH pulse and LH secretion, as kisspeptin can excite GnRH neurons and NKB work as stimulatory factor and dynorphin as inhibitory factor of kisspeptin production, then modulate downstream GnRH secretion (18). Furthermore, KNDy neurons are involved in the negative feedback regulation of estrogen to the HPO axis through estrogen receptor (19). On the contrary, kisspeptin neurons in AVPV/PeN are implicated in the formation of estradiol-induced LH surge before ovulation (20). In summary, kisspeptin is an important regulator in the circular regulation from brain to gonads.

It has long been reported that hypothalamic kisspeptin levels are increased in PCOS patients and PCOS animal models, which is the master contributor to increased LH pulse secretion (19, 21). While Panidis et al. found that serum kisspeptin level in PCOS patients was not significantly increased, and even decreased when compared to control (22). As kisspeptin mainly takes effect in the hypothalamus, so serum kisspeptin may be less related to the activation of GnRH neurons and LH pulse synthesis. Apart from NKB and dynorphin, a number of metabolic regulators also contribute to the modulation of kisspeptin neurons. Mice lacking both insulin and leptin receptors in POMC neurons displayed PCOS phenotype, including insulin resistance, elevated testosterone levels and reduced fertility (23); besides, hypothalamic POMC neurons send projections to kisspeptin neurons, indicating the possible role of POMC–kisspeptin pathway in PCOS pathogenesis.

Kisspeptin may also take effect in the ovaries. Gaytán et al. identified the expression of kisspeptin–GPR54 system genes in the human and rat ovary for the first time (24), and ovarian kisspeptin expression was positively regulated by gonadotropins for the fact that KISS1 gene expression increased after puberty onset. Furthermore, it seems that local kisspeptin system may directly modulate ovarian function (25). Recently, Blasco et al. compared the gene expression levels of KISS1/KISS1R, as well as TAC3 and TACR3 (encoding NKB and its G-protein coupled receptor NK3R respectively) in infertile patients and healthy control and found that defected fertility may be associated with the alteration of local KISS1/KISS1R expression in the ovaries (26). Besides, the kisspeptin/KISS1R and NKB/NK3R systems are decreased in PCOS mural granulosa cells and cumulus cells, indicating that abnormal ovarian kisspeptin and NKB may contribute to aberrant follicle development in PCOS patients (27). However, the specific molecular mechanism underlying the local effect of kisspeptin on PCOS ovarian function still needs to be discovered.

#### Galanin

Found in 1983, neuropeptide galanin is widely distributed in the brain and peripheral organs. Galanin signals through G-protein coupled receptor GAL1-3 which is expressed by ARC GnRH neurons, indicating that galanin is implicated in the modulation of GnRH. Besides, galanin is also implicated in the regulation of glucose metabolism and thermogenesis (28, 29), which makes it a molecular motif integrating metabolism and neuroendocrine-reproduction axis. However, the role galanin played in PCOS development remains unclear. Recently, Azin et al. explored the effect of galanin on estradiol valerate-induced PCOS rat. Intraperitoneal injection of galanin induced increased FSH levels and decreased LH and insulin levels, thus alleviating the metabolic disorders in PCOS rat. Furthermore, serum TNF-α and IL-6 levels were significantly increased in PCOS group, which was reversed with galanin treatment (29). Altinkaya compared serum galanin levels in 44 women with PCOS and 44 age-matched controls and found that women with PCOS were characterized by lower galanin levels than controls (30), indicating that supplementation of galanin may be a new therapeutic approach for PCOS, which still needs more evidence to support.

### Neurotransmitters

Neurotransmitters, especially GnRH-regulatory neurotransmitters can be important in the pathogenesis of PCOS (31). Although GABA is usually considered as an inhibitory neurotransmitter in the brain, compelling evidence suggests its stimulatory effect on GnRH neurons (32). It is reported that the number of GABAergic synapses onto KNDy neurons increased significantly in prenatal testosterone exposed ewes, which means that GABA can activate KNDy neurons as well as GnRH neurons, thus elevating the pulse frequency of GnRH and LH in PCOS (33). Furthermore, higher cerebrospinal fluid GABA levels are observed in women with PCOS, along with increased circulating levels of E2 and T (34). Silva et al. investigated the effect of acute stimulation and chronic activation of GABA neurons on LH synthesis and found that both ways can increase LH levels. Besides, chronic activation of GABA neurons induces PCOS-like phenotypes in mice, including high circulating testosterone levels, irregular estrous cycle, and decreased corpora lutea number (32). In addition, the hypothalamic GABA neurons showed less expression of progesterone receptor in PCOS mice, which impairs GABA-mediated feedback effect of progesterone on GnRH neurons (35).

## Ovarian Hormones Modulate the Action of GnRH Neurons

PCOS patients are characterized by aberrant sex hormone levels; hyperandrogenemia is the most consistent characterization observed in women with PCOS. Besides, aromatase is inhibited in PCOS granulosa cell, leading to aberrant estrogen levels. Abnormally increased AMH levels are also observed in PCOS patients (36); all these sex hormones substantially affect neuronal activity in the brain, which forms a vicious circle, thus promoting ovarian dysfunction and reproductive disorders in women with PCOS.

### Androgen

Acting *via* AR, androgen is involved in both intra- and extra-ovarian mechanisms of PCOS pathogenesis. It is reported that AR is hyperactivated in the hypothalamus, ovary, skeletal muscle, and adipose cells in women with PCOS (37), which means the action of androgen in those tissues may mediate PCOS development. Mounting evidence identifies that androgen is implicated in manipulating hypothalamic GnRH neuron activity, as increased LH pulse frequency and amplitude are observed in both PCOS patients and PCOS animal models. In addition, DHT treatment in ovariectomized and estradiol-treated mice increased the connectivity of GABAergic neurons and GnRH neurons, which was inhibited by progesterone treatment (38), indicating the possible role of androgen in modulating the negative feedback regulation of progesterone on GnRH neurons, and then increasing GnRH pulse frequency and amplitude.

In addition, Kiss1 gene expression and LH pulse frequency are increased in PNA mice, which means that KNDy neurons can be another central target of androgen. ARKO mice exhibit impaired GnRH synthesis pattern and decreased Kiss1 gene expression in anteroventral periventricular nucleus, leading to deficient preovulatory estrogen and LH surge, which is consistent with the reduction of ovarian corpora lutea numbers in ARKO mice (39). To investigate the precise mechanism of AR-mediated androgen action on GnRH synthesis, Cheng et al. generated neuron-specific AR knockout mice (NeurARKO) and found similar neuroendocrine feature to ARKO mice. In terms of ovarian follicle dynamics, increased follicle atresia and reduced ovulation were found in NeurARKO mice (40). Although the effect of androgen on peripheral tissue and organs is implicated in PCOS pathogenesis, the effect of androgen on central nervous system plays a pivotal role in PCOS development, because there is little difference in GnRH synthesis pattern and ovarian follicle dynamics between ARKO mice and NeurARKO mice.

On the other hand, prenatal androgen treated animal models are more often used for research investigating neuroendocrinal pathogenesis in PCOS, considering the high intrauterine androgen environment during pregnancy in women with PCOS, indicating that androgen and androgen activated GnRH synthesis may drive PCOS development since embryo, and this effect consistently exists till adulthood, leading to PCOS in offspring. So, the modulation of androgen on GnRH neurons during pregnancy can be quite important in PCOS development.

### Anti-Müllerian Hormone

It is well known that AMH facilitates the modulation of ovarian follicle growth and now is widely used as a predictor of ovarian reserve in clinical work. In women with PCOS, AMH levels are increased due to accumulation of small antral follicles in the ovary (41). On the other hand, AMH can decrease FSH receptor and aromatase expression in granulosa cells (42), which impairs follicle growth and leads to follicular arrest, thus forming a vicious cycle. Apart from the effect on ovary, AMH also takes effect on HPO axis. AMH has high affinity to AMH receptor AMHR2, both AMH and AMHR2 are expressed in GnRH neurons (43, 44). Cimino et al. found that AMH can induce LH secretion *via* stimulating hypothalamic GnRH neurons directly, which needs further research to confirm in PCOS. Recently, Tata et al. found that serum AMH levels are significantly elevated in pregnant women with PCOS than in control women, and they use PAMH mice to investigate the effect of elevated intrauterine AMH levels on neuroendocrine and reproductive function in offspring. It turns out that PAMH mice have significantly higher LH pulse frequency and circulating testosterone levels, as well as longer ano-genital distance which reflects androgenic impregnation, while prenatal GnRH antagonist treatment can reverse the neuroendocrine and reproductive abnormalities in PAMH mice. Besides, GnRH antagonist treatment can normalize the neuroendocrine and reproductive disorders in adult PAMH mice (45), which further confirmed the stimulatory effect of AMH on GnRH neurons.

In conclusion, acting as a stimulator of hypothalamic GnRH neurons, AMH treatment, both pre- and postnatal treatments, can increase LH pulse frequency and induce reproductive disorder like PCOS, and this provides new evidence for the therapeutic effect of GnRH antagonist in women with PCOS.

## Metabolic Regulation of GnRH Synthesis in PCOS

### Insulin Resistance

Insulin resistance plays a pivotal role in the pathogenesis of PCOS, the direct consequence of which is abnormally elevated insulin levels. According to human and animal studies concerning the effect of insulin in PCOS development, insulin is considered as a co-effector of gonadotropins. Insulin can promote testosterone biosynthesis in human ovarian theca cell and reduce SHBG production (46), thus contributing to hyperandrogenism in women with PCOS. In addition, insulin can stimulate LH secretion directly (47), leading to aberrant reproductive function in PCOS. As mentioned before, hypothalamic POMC neurons express both insulin receptor and leptin receptor, and knock-out of insulin receptor and leptin receptor in POMC neurons induced PCOS phenotype, indicating the insulin and leptin can be powerful regulators of both kisspeptin and POMC neurons, which further promote PCOS development (23).

Leptin plays a vital role in the central regulation of food intake and energy expenditure, as well as glucose metabolism, which makes leptin an important adipose-derived hormone in promoting insulin resistance. Besides, leptin may contribute to the effect of central and peripheral insulin resistance on obesity; these two pathophysiological changes often work together to promote metabolic disorders in metabolic diseases (48, 49). PCOS patients can be described as leptin resistance, as circulating leptin levels are higher in PCOS patients than in control, which is related to IR in PCOS patients (50, 51), suggesting that leptin may be implicated in the pathogenesis of PCOS. However, down-regulated hypothalamic leptin receptor expression is observed in PNA mice; additionally, leptin receptor is co-localized with kisspeptin and NKB in the ARC of PNA mice, indicating the possible interaction between leptin and kisspeptin/NKB. Further studies show that central administration of leptin can significantly stimulate hypothalamic *Kiss1* gene expression, as well as LH secretion, which can be suppressed by pretreatment with kisspeptin antagonist Kp-234 (52). This study provides a new insight into PCOS pathogenesis by shedding lights on the stimulatory effect of increased leptin levels on KNDy neurons and LH secretion.

### Obesity

Obesity is another manifestation of metabolic syndrome in PCOS, while the relationship between obesity and PCOS is much more complex. Firstly, the vicious circle of mutual reinforcing relationship between obesity and insulin and leptin resistance plays an important role in PCOS pathogenesis (49, 53). Hypothalamic leptin resistance has been identified to increase weight gain; at the same time, enhanced leptin secretion by adipocytes further contributes to induce leptin resistance, thus promoting PCOS development (54). In addition, growing piece of evidence show that kisspeptin also mediate obesity-related effect on reproductive function. To figure out the effect of bariatric surgery (a kind of treatment for PCOS patients to lose weight) on hypothalamic kisspeptin expression, Wen et al. performed sleeve gastrectomy (SG) for PCOS rat. After SG, metabolic disorders in PCOS rat including impaired glucose tolerance, decreased insulin sensitivity, and adiponectin levels are reversed, which is accompanied by decreased KISS1 gene expression in ARC, indicating that over-activated kisspeptin neurons can mediate metabolic regulation of central nervous system, then contribute to metabolic induced reproductive dysfunction in PCOS (55). Interestingly, Wen et al. also found that after SG, there is no significant loss of body weight in PCOS rat, which means that body weight may not implicate in the regulation of KISS1 gene expression. A recent study further confirmed this hypothesis, for the difference of kisspeptin levels between normal-weight PCOS patients and over-weight PCOS patients is insignificant (56). Thus, alternative obesity-related pathway mediating central control of reproductive function needs to be explored.

Growing pieces of evidence suggest that activated sympathetic nervous system takes part in PCOS and obesity pathogenesis (57, 58). Interaction between sympathetic nervous activation and obesity also implicates in PCOS pathogenesis, while the underlying mechanism remains unclear. Adiponectin is an adipocytokine known to play a pivotal role in the regulation of insulin sensitivity, as well as the control of ovarian follicle growth and early embryo development (34, 59). A systemic review identified the lower circulating adiponectin levels in women with PCOS, which are related to IR but not to BMI (60), indicating that there is little correlation between obesity and adiponectin levels in PCOS patients. Instead of the relationship with IR, Shorakae et al. mainly focus on adiponectin’s regulation of sympathetic function (61). Muscle sympathetic nerve activity is increased in women with PCOS, along with decreased high molecular weight adiponectin levels. That is to say, similar to other diseases, sympathetic stimulation can reduce adiponectin levels in PCOS. Adiponectin may contribute to PCOS development *via* regulation of insulin resistance and sympathetic nerve activity. Recently, Heras et al. found that increased hypothalamic ceramide levels were involved in an alternative PVN-ovarian sympathetic innervation pathway, rather than the classical GnRH dependent pathway, thus promoting obesity-induced precious puberty (62), which provided new insights for the effect of obesity and sympathetic nervous system activation in PCOS pathogenesis.

Overall, obesity is common in PCOS patient, and lifestyle modifications including weight reduction are the primary treatment to improve metabolic dysfunction and infertility in women with PCOS. While the exact role of obesity or obesity related sympathetic activation in PCOS development still needs to be explored.

## Impact of Intestinal Microbiome on PCOS Pathophysiology

The imbalance of gut microenvironment is closely related to the pathogenesis of different kinds of diseases (63). In addition, the crosstalk between gut and brain has long been appreciated (64). This part mainly focuses on the possible mechanism of gut hormones and gut microbiota disturbance affecting the pathogenesis of PCOS through the gut–brain axis.

### Gut Hormone

Gut hormones are vital mediators in bidirectional communication of the gut–brain axis and are implicated in different kinds of metabolic diseases. GLP-1 is mainly synthesized by intestinal L cells and acts through G protein-coupled GLP-1R which is found in many tissues in the human body, including brain and reproductive system. A growing body of evidence shows that GLP-1 is now widely used in women suffering PCOS, and the clinical effects of GLP-1 include improvement of ovulation, elevation of menstrual frequency, and promotion of pregnancy rate in women with PCOS (65–67). In terms of sex hormone, liraglutide decreased free testosterone and androstenedione levels and increased SHBG levels in women with PCOS (66). Besides, GLP-1 is recommended as a therapeutic option for obese women with PCOS for its significant weight loss effect. Although GLP-1 has been widely recognized in PCOS treatment, the underlying mechanism remains vague now.

The interaction between GLP-1 and hypothalamic GnRH neurons has long been discovered. Outeiriño-Iglesias et al. investigated the effect of GLP-1 on LH synthesis and found that acute administration of GLP-1significantly increases the amplitude of LH surge before ovulation in adult rats, while GLP-1R agonist Exendin-4 can block the stimulatory effect of GLP-1 on LH synthesis (68). Interestingly, liraglutide, a GLP-1R agonist, is able to depolarize ARC kisspeptin neurons directly but cannot reverse the inhibition of ARC kisspeptin neurons after 48 h fast (69), which means that GLP-1 cannot maintain LH synthesis alone. In addition, GLP-1 can activate GnRH neurons directly *via* GLP-1R, as well as modulation of stimulatory presynaptic GABAergic inputs to GnRH neurons (70). Overall, GLP-1 is identified as a stimulator of GnRH neurons *via* modulation of kisspeptin neurons and GABAergic neurons, indicating that GLP-1 may take part in the modulation of GnRH and LH synthesis, thus contributing to PCOS development.

Except for the regulation of metabolism and LH synthesis, GLP-1 is also a vital mediator of the influence of gut microbiota on host. Gut microbiota fragmentation of non-digestible carbohydrates is known to promote glucose metabolism, increase satiety, and reduce food intake, thus maintaining energy balance (71, 72). Additionally, GLP-1 secretion was augmented by supplementation of dietary fibers, and this process was mediated by SCFAs: as the metabolites of dietary fibers, SCFAs promoted GLP-1 secretion *via* receptors (GPR-41/43) expressed by intestinal enteroendocrine L cells (73–75). So GLP-1 may play an important role in gut microbiota dysbiosis related diseases. Hwang et al. found that antibiotics-induced reduction of Firmicutes and Bacteroidetes significantly augmented serum GLP-1 levels and GLP-1 expression, thus improving insulin resistance in diet-induced-obesity mice (76). Overall, GLP-1 participated in PCOS pathogenesis through multiple ways; therapeutics targeting GLP-1 secretion can be promising for PCOS treatment.

### Gut Microbiota Dysbiosis

Considering the close relationship between gut microbiota and host diseases, the relationship between gut microbiota and PCOS is now attracting more and more attention. It is reported that alpha diversity in PCOS patient is decreased, which may be related to reproductive dysfunction and metabolic dysregulation (8). On the contrary, aberrant sex hormone in PCOS patients may have an impact on gut microbiota as well, which makes it complex to find out the true role of microbiota in PCOS development.

It has been widely reported that the gut microbiota is capable of producing neurotransmitters including dopamine, noradrenaline, serotonin, and GABA (77). As described before, GABA is a powerful neurotransmitter that activates GnRH neurons and increases GnRH pulse frequency and amplitude, thus promoting PCOS development. Furthermore, it’s reported that high-fat diet leads to reduced levels of Bacteroides, which reduce GABA levels in rat prefrontal cortex and alleviate depressive-like behavior (78). This indicates that the gut microbiota may modulate neurotransmitter levels in the central nervous system and then change the function of downstream neurons and the emotional state of host. While evidence indicating that microbial-derived neurotransmitter act directly on central neurons is still lacking, the effect of microbial-derived GABA on PCOS neuroendocrine disorder remains unclear. Qi et al. found that the abundance of *B. vulgatus* is increased in PCOS patients (9), which is associated with aberrant hormone levels like high androgen, LH levels, and increased LH/FSH ratio, and this is usually considered as the result of activating GnRH neurons. The relationship between *B. vulgatus* and GABA levels has not been researched; however, GABA may be a dot that connects *B. vulgatus* and neuroendocrine disorders in PCOS. Besides, there are some kinds of GABA-producing bacteria found to be increased and positively correlated with serum LH levels and LH/FSH ratio, which provide a perspective to understand the underlying mechanism of gut–brain axis in PCOS development (79).

Gut microbial metabolites and microbiota-regulated metabolic process also play a vital role in the gut–brain axis. As the most examined gut microbial metabolites, SCFAs have been implicated in maintaining intestinal barrier integrity and host immune homeostasis (64, 80). In addition, SCFAs are involved in gut–brain crosstalk; supplementation of SCFAs can alleviate increased blood–brain barrier permeability in GF mouse and modulate histone acetylation in the cortex of GF mouse. Although it seems like SCFAs play a negative role in the development of Parkinson’s disease (81), SCFAs are more thought to be beneficial to host homeostasis. Zhang et al. found that there is a significant decrease in PCOS patients’ intestinal SCFA levels, while supplying probiotic *Bifidobacterium lactis* V9 can rescue the decreased SCFA levels in PCOS patients (82). Besides, the colonization of *Bifidobacterium lactis* V9 is related to decreased LH and LH/FSH levels. Furthermore, they explored the correlation among the identified MGS, metabolic parameters, SCFAs, and sex hormones and found that the colonization of *Bifidobacterium lactis* V9 promotes the growth of SCFA-producing microbiotas, thus promoting PYY and ghrelin secretion, which may act on hypothalamus GnRH neurons and mediate the beneficial effect of microbial derived SCFAs in alleviating neuroendocrine disorders of PCOS patients.

As a class of microbial metabolites, bile acids are attracting more and more attention for their serious impact on regulating host immune cell function and modulating host metabolic status, as well as brain function. PCOS is known as a metabolic syndrome with reproductive disorder, aberrant bile acid metabolism may also participate in PCOS development, as Zhang et al. have reported that increased circulating conjugated primary bile acid levels are positively correlated with hyperandrogenemia in women with PCOS (83). On the other hand, Qi et al. found that serum and intestinal secondary bile acid GDCA and TUDCA levels are significantly decreased in PCOS patients (9). Furthermore, GDCA and TUDCA levels were negatively correlated with *B. vulgatus* and bile salt hydrolase (*bsh*) gene abundance, both of which are increased in women with PCOS. So abnormal bile acid metabolism induced by gut microbiota disturbance can be a key segment in PCOS development. In terms of the underlying mechanism, microbial-derived bile acids can activate ILC3s and their secretion of IL-22, thus improving insulin resistance in PCOS patients. Moreover, supplementation of GDCA can decrease serum testosterone levels in DHEA-induced PCOS-like mouse, indicating that bile acid may act through multiple ways to improve PCOS, so bile acids may act through the hypothalamus to regulate sex hormone levels directly.

## Conclusion

**Figure 1:**
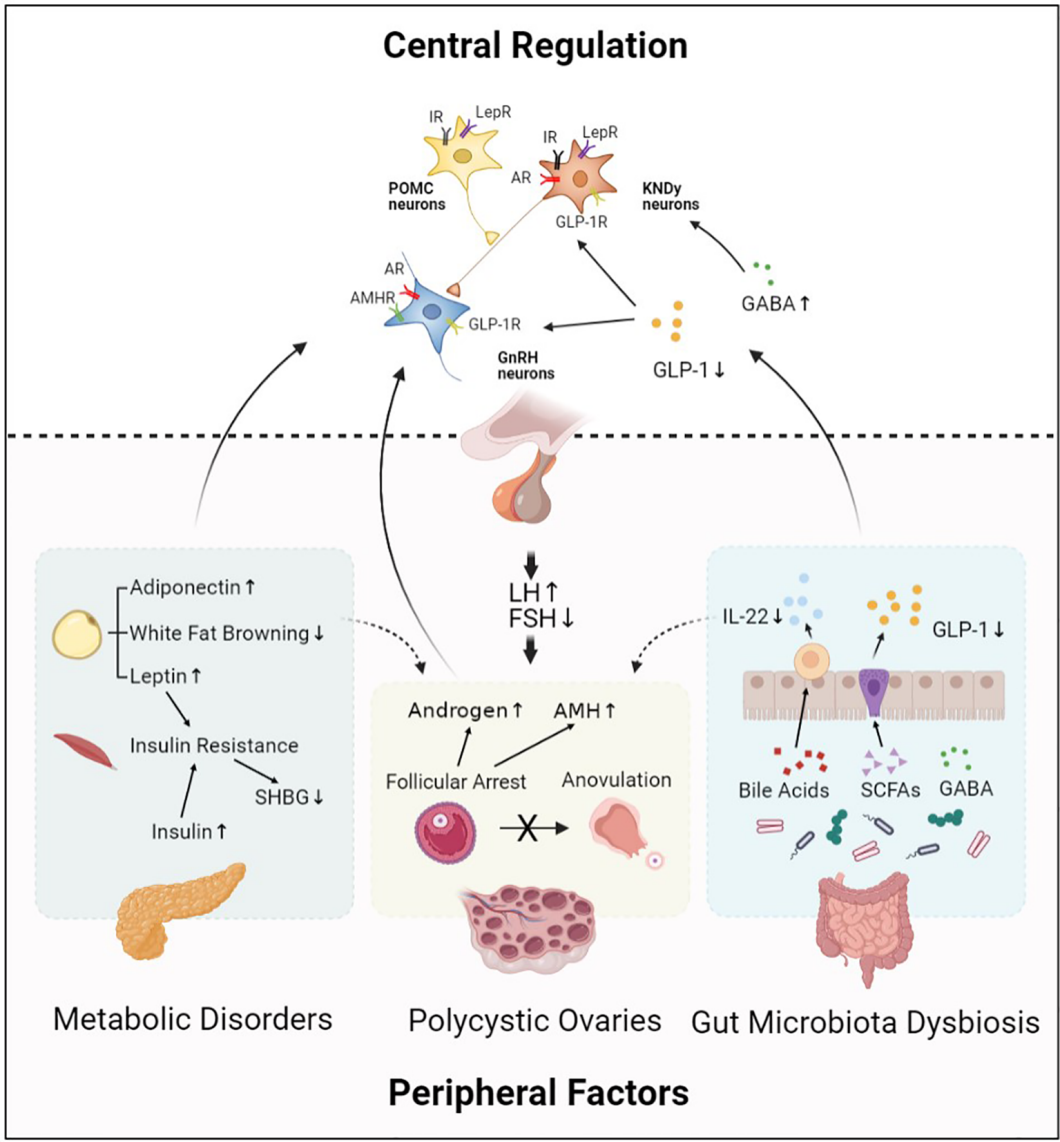
Central regulation of PCOS. Hypothalamic GnRH pulse mediates regulation of LH and FSH synthesis, which plays an important role in PCOS pathophysiology. This process is modulated by central regulators including KNDy neurons, POMC neurons, and neurotransmitters. In addition, peripheral factors including abnormal ovarian hormone levels, metabolic disorders, and gut microbiota dysbiosis also contribute to PCOS development by acting on their receptors expressed in hypothalamic neurons. Moreover, central regulators and peripheral factors interact with each other and form an abnormal neuronal–reproductive–metabolic circuit, thus promoting PCOS development. GnRH, Gonadotropin-releasing hormone; KNDy neurons, kisspeptin/NKB/dynorphin A neurons; POMC neurons, Pro-opiomelanocortin neurons; GABA, *γ*-aminobutyric acid; GLP-1, Glucagon-like peptide-1; IR, Insulin receptor; LepR, Leptin receptor; AR, Androgen receptor; GLP-1R, Glucagon-like peptide-1 receptor; AMHR, Anti-Müllerian hormone receptor; LH, Luteinizing hormone; FSH, Follicle stimulating hormone; AMH, Anti-Müllerian hormone; SHBG, Sex hormone-binding globulin; IL-22, Interleukin 22; SCFAs, Short-chain fatty acids.

PCOS is the most common endocrine disorder in women of reproductive age, aberrant HPO axis is at the center of PCOS pathogenesis (**Figure 1**). Kisspeptin and GABA are involved in the upstream regulation of GnRH neurons activity, which forms the final common pathway of central regulation of PCOS development. The direct stimulatory effect of androgen and AMH on GnRH neurons is considered as potential key mechanism involved in the origins of the neuroendocrine dysfunctions of PCOS. Besides, metabolic disorders including insulin resistance and leptin resistance also contribute to abnormalities of GnRH neurons in PCOS. However, obesity is more likely to be involved in the sympathetic activation in PCOS development. Gut hormone GLP-1 has long been recommended as treatment for obese women with PCOS; it is also identified as a stimulator of GnRH neurons *via* modulation of kisspeptin neurons and GABAergic neurons. Moreover, gut microbial derived neurotransmitter GABA may take effect in hypothalamic GnRH neurons and thus promoting PCOS development. Metabolites of the gut microbiota, including SCFAs and bile acids, are effective regulators of GnRH neurons function.

Considering the vital role HPO axis played in PCOS pathogenesis, therapeutics targeting the HPO axis can be effective. Kisspeptin peptides kisspeptin-10 and kisspeptin-54 can increase LH levels in healthy women to promote ovulation (84, 85). With longer terminal half-life time, MVT-602, a novel KISS1R agonist, has longer duration of kisspeptin targeted action of stimulating GnRH synthesis and sustaining peak GnRH levels, consequently promoting LH synthesis in women with PCOS. Besides, estradiol pretreatment before administration of MVT-602 can increase both LH and FSH peak levels to that observed in preovulatory follicular phase, which means that supplementation of estradiol and MVT-602 is a promising treatment for *in vitro* fertilization of PCOS women. It seems that this still belongs to symptomatic treatment; while considering the central role of GnRH in PCOS pathogenesis, therapeutics targeting GnRH neurons can improve endocrine disorders, which further improves metabolic disorders and gut microbiota dysbiosis in PCOS.

Therapeutics improving metabolic disorders including sensitization of tissues to insulin and bariatric surgery to lose weight can in turn influence GnRH and sex hormone secretion, thus alleviating PCOS. Although the way that peripheral nervous system is implicated in PCOS pathogenesis remains unclear, reduced sympathetic activity is observed in heat-treated obese PCOS women, as well as decreased cardiovascular risk profiles (86). In addition, acupuncture with electrical stimulation also reduces endocrine and reproductive dysfunction in women with PCOS by modulating sympathetic activity (87). However, further research is needed to clarify the underlying mechanism.

Treatment targeting the gut microbiota is an emerging therapeutic for metabolic diseases. It is reported that the gut microbiota *A. muciniphila* increased thermogenesis of brown adipocytes and GLP-1 secretion in HFD mice, thus improving glucose homeostasis (88). High-fiber diet elevated GLP-1 levels in patients with type 2 diabetes *via* promoting the growth of SCFA-producing gut microbiota, and finally improved glucose regulation (89). Fecal bacteria transplantation shows great potential for metabolic disease treatment. A double-blind study was performed to figure out the effect of FMT obese patients. Patients who received FMT capsules presented bile acid profiles comparable to those of lean people (90). The gut microbiota-mediated effect is not just in the gut. Probiotics supplementation increased colonic GLP-1 levels and cerebral GLP-1 receptor expression in mice model of Parkinson disease, indicating that probiotics supplementation could improve cerebral function through the gut microbiota–gut–brain axis (91). Gut microbiota related GABA was implicated in the development of seizure and schizophrenia (92, 93). So, the therapeutic effect of gut microbial-derived metabolites and probiotics in various diseases has been confirmed. Although the effect of gut microbiota-related treatment in women with PCOS still needs further study, fecal bacteria transplantation and supplementation of probiotics all show great potential for PCOS treatment.

In conclusion, the mechanism underlying PCOS pathogenesis is complicated, so is the relationship between neuroendocrine defects, metabolic disorders, and intestinal microbiota dysbiosis in PCOS pathophysiology. In terms of the causal relationship between the central defects and peripheral factors implicated in PCOS pathogenesis, one supposes that reproductive and metabolic disorders lead to defects in the brain of PCOS women, since the effect of exposure to excessive androgen and insulin during pregnancy cannot be ignored for the fact that PCOS mouse model can be established only by androgen injection to pregnant mouse. The other favors the idea that abnormal activation of GnRH neurons is the causality of reproductive and metabolic disorders, as increased GABAergic wiring to hypothalamic GnRH neurons occurred before PCOS mice exhibited disease phenotypes (87). It seems to make sense because of the significant therapeutic effect of inhibiting GnRH neurons in PCOS. But it is still unclear when and how the over-activation of GnRH neurons is formed in the brain, which needs further research studies. Actually, it’s more accurate to say that it is the abnormal neuroendocrine–reproductive–metabolic circuit that plays an important role in the pathogenesis of PCOS.

As a rising research field, the gut microbiota is implicated in the development of various diseases through the gut–brain axis, gut-liver axis, *etc.* While direction of the regulation of these pathways remains unclear, one takes the view that the change of the gut microbiota is the cause of metabolic disorders and neuroendocrine defects, for gut microbiota transplantation can transfer donor phenotypes to recipients (94). The other takes the different view that it’s just association rather than causality because the composition of gut microbiota is closely related to dietary history, and the gut microbiota transplantation experiments are mainly applied in germ-free mouse which exhibit different intestinal function. However, microbial fingerprinting model was established based on long-term investigation of the compositional and genomic stability of gut microbes, indicating that the gut microbiota composition and metabolites may influence host phenotype in a stable and chronic way. The debate may continue, and more experiments are needed (95).

PCOS is a heterogeneous and complex disorder in women of reproductive age, the pathophysiology of which is not clearly understood yet. Classic theory presumed that the abnormal activation of hypothalamus GnRH neurons and excessive ovarian androgen synthesis are the core of pathogenic mechanism in PCOS. With research studies are getting deeper, the important role of metabolic disorders and gut microbiota dysbiosis in PCOS pathogenesis has been identified. To some extent, reproductive and metabolic disorders and gut microbiota dysbiosis contribute to the impairment of local ovarian function, and their effect on activating GnRH synthesis drives the development of PCOS. However, it’s hard to define the accurate onset time and location of this complex syndrome. Abnormal exposure to AMH, androgen, or insulin during pregnancy can promote PCOS development, and the underlying mechanism of which lies in the hyper-secretion of GnRH. Therefore, primary defects in the brain may be the direct cause of PCOS; at the same time, metabolic disorders, local ovarian hormone and gut microbiota dysbiosis can act on GnRH neurons, thus cooperatively promoting PCOS development. Overall, these important insights provide us with a new perspective that the brain plays a key role in the origin of PCOS and opens new avenues for investigating therapeutic interventions for women with PCOS.

## Author Contributions

All authors listed have made a substantial, direct and intellectual contribution to the work, and approved it for publication.

## Funding

This work was supported by the National Key Research and Development Program of China (2018YFC1003200, 2018YFC1003900), National Natural Science Foundation of China (82022028, 81730038), Key Clinical Projects of Peking University Third Hospital (BYSYZD2019020), and CAMS Innovation Fund for Medical Sciences (2019-I2M-5-001).

## Conflict of Interest

The authors declare that the research was conducted in the absence of any commercial or financial relationships that could be construed as a potential conflict of interest.

## Abbreviations

PCOS, Polycystic ovary syndrome; GnRH, Gonadotropin-releasing hormone; LH, Luteinizing hormone; PCOM, Polycystic ovary morphology; IR, Insulin resistance; GWAS, Genome-wide association studies; FSH, Follicle stimulating hormone; AMH, Anti­Müllerian hormone; IL-22, Interleukin-22; HPO axis, Hypothalamus–pituitary–ovary axis; CNS, Central nervous system; IGF-1, Insulin-like growth factor 1; ARC, Arcuate nucleus; AVPV/PeN, Anteroventral periventricular nucleus/periventricular nucleus continuum; NKB, Neurokinin B; KNDy, Kisspeptin/NKB/dynorphin A; POMC, Pro-opiomelanocortin; GABA, *γ*-aminobutyric acid; E2, Estradiol; T, Testosterone; AR, Androgen receptor; PNA, Prenatal androgen treated; ARKO, AR knockout; PAMH, Prenatal AMH treated; SHBG, Sex hormone-binding globulin; PVN, Paraventricular nucleus of hypothalamus; GLP-1, Glucagon-like peptide-1; GLP-1R, GLP-1 receptor; SCFAs, Short-chain fatty Acids; PYY, Peptide YY; GF, Germ-free; GDCA, Glycodeoxycholic acid; TUDCA, Tauroursodeoxycholic acid; ILC3s, Group 3 innate lymphoid cells.

## References

[1] Norman RJ Dewailly D Legro RS Hickey TE. Polycystic Ovary Syndrome. Lancet (2007) 370(9588):685–97. 10.1016/S0140-6736(07)61345-2 17720020

[2] Azziz R Carmina E Chen Z Dunaif A Laven JS Legro RS. Polycystic Ovary Syndrome. Nat Rev Dis Primers (2016) 2:16057. 10.1038/nrdp.2016.57 27510637

[3] Jayasena CN Franks S. The Management of Patients With Polycystic Ovary Syndrome. Nat Rev Endocrinol (2014) 10(10):624–36. 10.1038/nrendo.2014.102 25022814

[4] McAllister JM Legro RS Modi BP Strauss JF 3rd. Functional Genomics of PCOS: From GWAS to Molecular Mechanisms. Trends Endocrinol Metab (2015) 26(3):118–24. 10.1016/j.tem.2014.12.004 PMC4346470 25600292

[5] Shi Y Zhao H Shi Y Cao Y Yang D Li Z. Genome-Wide Association Study Identifies Eight New Risk Loci for Polycystic Ovary Syndrome. Nat Genet (2012) 44(9):1020–5. 10.1038/ng.2384 22885925

[6] Dumesic DA Oberfield SE Stener-Victorin E Marshall JC Laven JS Legro RS. Scientific Statement on the Diagnostic Criteria, Epidemiology, Pathophysiology, and Molecular Genetics of Polycystic Ovary Syndrome. Endocr Rev (2015) 36(5):487–525. 10.1210/er.2015-1018 26426951 PMC4591526

[7] Abbott DH Nicol LE Levine JE Xu N Goodarzi MO Dumesic DA. Nonhuman Primate Models of Polycystic Ovary Syndrome. Mol Cell Endocrinol (2013) 373(1-2):21–8. 10.1016/j.mce.2013.01.013 PMC3683573 23370180

[8] Thackray VG. Sex, Microbes, and Polycystic Ovary Syndrome. Trends Endocrinol Metab (2019) 30(1):54–65. 10.1016/j.tem.2018.11.001 30503354 PMC6309599

[9] Qi X Yun C Sun L Xia J Wu Q Wang Y. Gut Microbiota-Bile Acid-Interleukin-22 Axis Orchestrates Polycystic Ovary Syndrome. Nat Med (2019) 25(8):1225–33. 10.1038/s41591-019-0509-0 PMC7376369 31332392

[10] Qi X Yun C Liao B Qiao J Pang Y. The Therapeutic Effect of interleukin-22 in High Androgen-Induced Polycystic Ovary Syndrome. J Endocrinol (2020) 245(2):281–9. 10.1530/JOE-19-0589 32163914

[11] Gilling-Smith C Willis DS Beard RW Franks S. Hypersecretion of Androstenedione by Isolated Thecal Cells From Polycystic Ovaries. J Clin Endocrinol Metab (1994) 79(4):1158–65. 10.1210/jc.79.4.1158 7962289

[12] Taylor AE McCourt B Martin KA Anderson EJ Adams JM Schoenfeld D. Determinants of Abnormal Gonadotropin Secretion in Clinically Defined Women With Polycystic Ovary Syndrome. J Clin Endocrinol Metab (1997) 82(7):2248–56. 10.1210/jcem.82.7.4105 9215302

[13] Cara JF Fan J Azzarello J Rosenfield RL. Insulin-Like Growth Factor-I Enhances Luteinizing Hormone Binding to Rat Ovarian Theca-Interstitial Cells. J Clin Invest (1990) 86(2):560–5. 10.1172/JCI114745 PMC296761 2384603

[14] Herbison AE. Control of Puberty Onset and Fertility by Gonadotropin-Releasing Hormone Neurons. Nat Rev Endocrinol (2016) 12(8):452–66. 10.1038/nrendo.2016.70 27199290

[15] Aparicio SA. Kisspeptins and GPR54–the New Biology of the Mammalian GnRH Axis. Cell Metab (2005) 1(5):293–6. 10.1016/j.cmet.2005.04.001 16054076

[16] Zhang C Bosch MA Qiu J Ronnekleiv OK Kelly MJ. 17beta-Estradiol Increases Persistent Na(+) Current and Excitability of AVPV/PeN Kiss1 Neurons in Female Mice. Mol Endocrinol (2015) 29(4):518–27. 10.1210/me.2014-1392 PMC4399275 25734516

[17] Kauffman AS Clifton DK Steiner RA. Emerging Ideas About Kisspeptin- Gpr54 Signaling in the Neuroendocrine Regulation of Reproduction. Trends Neurosci (2007) 30(10):504–11. 10.1016/j.tins.2007.08.001 17904653

[18] Cheng G Coolen LM Padmanabhan V Goodman RL Lehman MN. The Kisspeptin/Neurokinin B/Dynorphin (Kndy) Cell Population of the Arcuate Nucleus: Sex Differences and Effects of Prenatal Testosterone in Sheep. Endocrinology (2010) 151(1):301–11. 10.1210/en.2009-0541 PMC2803147 19880810

[19] Tang R Ding X Zhu J. Kisspeptin and Polycystic Ovary Syndrome. Front Endocrinol (Lausanne) (2019) 10:298. 10.3389/fendo.2019.00298 31156550 PMC6530435

[20] Navarro VM. Metabolic Regulation of Kisspeptin - the Link Between Energy Balance and Reproduction. Nat Rev Endocrinol (2020) 16(8):407–20. 10.1038/s41574-020-0363-7 PMC8852368 32427949

[21] Esparza LA Schafer D Ho BS Thackray VG Kauffman AS. Hyperactive LH Pulses and Elevated Kisspeptin and NKB Gene Expression in the Arcuate Nucleus of a PCOS Mouse Model. Endocrinology (2020) 161(4):1–15. 10.1210/endocr/bqaa018 PMC7341557 32031594

[22] Panidis D Rousso D Koliakos G Kourtis A Katsikis I Farmakiotis D. Plasma Metastin Levels are Negatively Correlated With Insulin Resistance and Free Androgens in Women With Polycystic Ovary Syndrome. Fertil Steril (2006) 85(6):1778–83. 10.1016/j.fertnstert.2005.11.044 16650418

[23] Hill JW Elias CF Fukuda M Williams KW Berglund ED Holland WL. Direct Insulin and Leptin Action on Pro-Opiomelanocortin Neurons is Required for Normal Glucose Homeostasis and Fertility. Cell Metab (2010) 11(4):286–97. 10.1016/j.cmet.2010.03.002 PMC2854520 20374961

[24] Gaytan F Gaytan M Castellano JM Romero M Roa J Aparicio B. KiSS-1 in the Mammalian Ovary: Distribution of Kisspeptin in Human and Marmoset and Alterations in KiSS-1 Mrna Levels in a Rat Model of Ovulatory Dysfunction. Am J Physiol Endocrinol Metab (2009) 296(3):E520–31. 10.1152/ajpendo.90895.2008 19141682

[25] Qi X Salem M Zhou W Sato-Shimizu M Ye G Smitz J. Neurokinin B Exerts Direct Effects on the Ovary to Stimulate Estradiol Production. Endocrinology (2016) 157(9):3355–65. 10.1210/en.2016-1354 27580802

[26] Blasco V Pinto FM Fernandez-Atucha A Gonzalez-Ravina C Fernandez-Sanchez M Candenas L. Female Infertility is Associated With an Altered Expression of the Neurokinin B/Neurokinin B Receptor and Kisspeptin/Kisspeptin Receptor Systems in Ovarian Granulosa and Cumulus Cells. Fertility Sterility (2020) 114(4):869–78. 10.1016/j.fertnstert.2020.05.006 32811673

[27] Blasco V Pinto FM Fernandez-Atucha A Prados N Tena-Sempere M Fernandez-Sanchez M. Altered Expression of the Kisspeptin/KISS1R and Neurokinin B/Nk3r Systems in Mural Granulosa and Cumulus Cells of Patients With Polycystic Ovarian Syndrome. J Assist Reprod Genet (2019) 36(1):113–20. 10.1007/s10815-018-1338-7 PMC6338604 30382469

[28] Fang P Yu M Shi M Bo P Zhang Z. Galanin Peptide Family Regulation of Glucose Metabolism. Front Neuroendocrinol (2020) 56:100801. 10.1016/j.yfrne.2019.100801 31705911

[29] Azin F Khazali H. Neuropeptide Galanin and its Effects on Metabolic and Reproductive Disturbances in Female Rats With Estradiol Valerate (Ev) - Induced Polycystic Ovary Syndrome (Pcos). Neuropeptides (2020) 80:102026. 10.1016/j.npep.2020.102026 32063381

[30] Altinkaya SO. Galanin and Glypican-4 Levels Depending on Metabolic and Cardiovascular Risk Factors in Patients With Polycystic Ovary Syndrome. Arch Endocrinol Metab (2021). 10.20945/2359-3997000000340 PMC10522184 33740336

[31] Ilie IR. Neurotransmitter, Neuropeptide and Gut Peptide Profile in PCOS-pathways Contributing to the Pathophysiology, Food Intake and Psychiatric Manifestations of PCOS. Adv Clin Chem (2020) 96:85–135. 10.1016/bs.acc.2019.11.004 32362321

[32] Silva MSB Desroziers E Hessler S Prescott M Coyle C Herbison AE. Activation of Arcuate Nucleus GABA Neurons Promotes Luteinizing Hormone Secretion and Reproductive Dysfunction: Implications for Polycystic Ovary Syndrome. Ebiomedicine (2019) 44:582–96. 10.1016/j.ebiom.2019.05.065 PMC6606966 31178425

[33] Porter DT Moore AM Cobern JA Padmanabhan V Goodman RL Coolen LM. Prenatal Testosterone Exposure Alters Gabaergic Synaptic Inputs to GnRH and KNDy Neurons in a Sheep Model of Polycystic Ovarian Syndrome. Endocrinology (2019) 160(11):2529–42. 10.1210/en.2019-00137 PMC6779074 31415088

[34] Richards JS Liu Z Kawai T Tabata K Watanabe H Suresh D. Adiponectin and its Receptors Modulate Granulosa Cell and Cumulus Cell Functions, Fertility, and Early Embryo Development in the Mouse and Human. Fertil Steril (2012) 98(2):471–9 e1. 10.1016/j.fertnstert.2012.04.050 22633650 PMC3523112

[35] Moore AM Prescott M Marshall CJ Yip SH Campbell RE. Enhancement of a Robust Arcuate GABAergic Input to Gonadotropin-Releasing Hormone Neurons in a Model of Polycystic Ovarian Syndrome. Proc Natl Acad Sci U S A (2015) 112(2):596–601. 10.1073/pnas.1415038112 25550522 PMC4299257

[36] Pellatt L Hanna L Brincat M Galea R Brain H Whitehead S. Granulosa Cell Production of Anti-Mullerian Hormone is Increased in Polycystic Ovaries. J Clin Endocrinol Metab (2007) 92(1):240–5. 10.1210/jc.2006-1582 17062765

[37] Walters KA. Role of Androgens in Normal and Pathological Ovarian Function. Reproduction (2015) 149(4):R193–218. 10.1530/REP-14-0517 25516989

[38] Sullivan SD Moenter SM. Gabaergic Integration of Progesterone and Androgen Feedback to Gonadotropin-Releasing Hormone Neurons. Biol Reprod (2005) 72(1):33–41. 10.1095/biolreprod.104.033126 15342358

[39] Cheng XB Jimenez M Desai R Middleton LJ Joseph SR Ning G. Characterizing the Neuroendocrine and Ovarian Defects of Androgen Receptor-Knockout Female Mice. Am J Physiol Endocrinol Metab (2013) 305(6):E717–26. 10.1152/ajpendo.00263.2013 23880317

[40] Walters KA Edwards MC Tesic D Caldwell ASL Jimenez M Smith JT. The Role of Central Androgen Receptor Actions in Regulating the Hypothalamic-Pituitary-Ovarian Axis. Neuroendocrinology (2018) 106(4):389–400. 10.1159/000487762 29635226

[41] Desforges-Bullet V Gallo C Lefebvre C Pigny P Dewailly D Catteau-Jonard S. Increased Anti-Mullerian Hormone and Decreased Fsh Levels in Follicular Fluid Obtained in Women With Polycystic Ovaries at the Time of Follicle Puncture for *In Vitro* Fertilization. Fertil Steril (2010) 94(1):198–204. 10.1016/j.fertnstert.2009.03.004 19361798

[42] Pellatt L Rice S Dilaver N Heshri A Galea R Brincat M. Anti-Mullerian Hormone Reduces Follicle Sensitivity to Follicle-Stimulating Hormone in Human Granulosa Cells. Fertil Steril (2011) 96(5):1246–51.e1. 10.1016/j.fertnstert.2011.08.015 21917251

[43] Cimino I Casoni F Liu X Messina A Parkash J Jamin SP. Novel Role for Anti-Mullerian Hormone in the Regulation of GnRH Neuron Excitability and Hormone Secretion. Nat Commun (2016) 7:10055. 10.1038/ncomms10055 26753790 PMC4729924

[44] Baarends WM van Helmond MJ Post M van der Schoot PJ Hoogerbrugge JW de Winter JP. A Novel Member of the Transmembrane Serine/Threonine Kinase Receptor Family is Specifically Expressed in the Gonads and in Mesenchymal Cells Adjacent to the Mullerian Duct. Development (1994) 120(1):189–97. 10.1242/dev.120.1.189 8119126

[45] Tata B Mimouni NEH Barbotin AL Malone SA Loyens A Pigny P. Elevated Prenatal Anti-Mullerian Hormone Reprograms the Fetus and Induces Polycystic Ovary Syndrome in Adulthood. Nat Med (2018) 24(6):834–46. 10.1038/s41591-018-0035-5 PMC6098696 29760445

[46] Nestler JE Jakubowicz DJ de Vargas AF Brik C Quintero N Medina F. Insulin Stimulates Testosterone Biosynthesis by Human Thecal Cells From Women With Polycystic Ovary Syndrome by Activating its Own Receptor and Using Inositolglycan Mediators as the Signal Transduction System. J Clin Endocrinol Metab (1998) 83(6):2001–5. 10.1210/jcem.83.6.4886 9626131

[47] Adashi EY Hsueh AJ Yen SS. Insulin Enhancement of Luteinizing Hormone and Follicle-Stimulating Hormone Release by Cultured Pituitary Cells. Endocrinology (1981) 108(4):1441–9. 10.1210/endo-108-4-1441 6781875

[48] Yadav A Kataria MA Saini V Yadav A. Role of Leptin and Adiponectin in Insulin Resistance. Clin Chim Acta (2013) 417:80–4. 10.1016/j.cca.2012.12.007 23266767

[49] Konner AC Bruning JC. Selective Insulin and Leptin Resistance in Metabolic Disorders. Cell Metab (2012) 16(2):144–52. 10.1016/j.cmet.2012.07.004 22883229

[50] Zheng SH Du DF Li XL. Leptin Levels in Women With Polycystic Ovary Syndrome: A Systematic Review and a Meta-Analysis. Reprod Sci (2017) 24(5):656–70. 10.1177/1933719116670265 27798245

[51] Pehlivanov B Mitkov M. Serum Leptin Levels Correlate With Clinical and Biochemical Indices of Insulin Resistance in Women With Polycystic Ovary Syndrome. Eur J Contracept Reprod Health Care (2009) 14(2):153–9. 10.1080/13625180802549962 19340711

[52] Yan X Yuan C Zhao N Cui Y Liu J. Prenatal Androgen Excess Enhances Stimulation of the GNRH Pulse in Pubertal Female Rats. J Endocrinol (2014) 222(1):73–85. 10.1530/JOE-14-0021 24829217

[53] Escobar-Morreale HF San Millan JL. Abdominal Adiposity and the Polycystic Ovary Syndrome. Trends Endocrinol Metab (2007) 18(7):266–72. 10.1016/j.tem.2007.07.003 17693095

[54] Zhao S Zhu Y Schultz RD Li N He Z Zhang Z. Partial Leptin Reduction as an Insulin Sensitization and Weight Loss Strategy. Cell Metab (2019) 30(4):706–19 e6. 10.1016/j.cmet.2019.08.005 31495688 PMC6774814

[55] Wen L Lin W Li Q Chen G Wen J. Effect of Sleeve Gastrectomy on Kisspeptin Expression in the Hypothalamus of Rats With Polycystic Ovary Syndrome. Obes (Silver Spring) (2020) 28(6):1117–28. 10.1002/oby.22795 PMC7317914 32347662

[56] Araujo BS Baracat MCP Dos Santos Simoes R de Oliveira Nunes C Maciel GAR Lobo RA. Kisspeptin Influence on Polycystic Ovary Syndrome-a Mini Review. Reprod Sci (2020) 27(2):455–60. 10.1007/s43032-019-00085-6 31919796

[57] Shorakae S Lambert EA Jona E Ika Sari C de Courten B Dixon JB. Effect of Central Sympathoinhibition With Moxonidine on Sympathetic Nervous Activity in Polycystic Ovary Syndrome-A Randomized Controlled Trial. Front Physiol (2018) 9eeloc . 10.3389/fphys.2018.01486 PMC6210452 30410448

[58] Larabee CM Neely OC Domingos AI. Obesity: A Neuroimmunometabolic Perspective. Nat Rev Endocrinol (2020) 16(1):30–43. 10.1038/s41574-019-0283-6 31776456

[59] Bohler H Mokshagundam S Winters SJ. Adipose Tissue and Reproduction in Women. Fertility Sterility (2010) 94(3):795–825. 10.1016/j.fertnstert.2009.03.079 19589523

[60] Toulis KA Goulis DG Farmakiotis D Georgopoulos NA Katsikis I Tarlatzis BC. Adiponectin Levels in Women With Polycystic Ovary Syndrome: A Systematic Review and a Meta-Analysis. Hum Reprod Update (2009) 15(3):297–307. 10.1093/humupd/dmp006 19261627

[61] Shorakae S Abell SK Hiam DS Lambert EA Eikelis N Jona E. High-Molecular-Weight Adiponectin is Inversely Associated With Sympathetic Activity in Polycystic Ovary Syndrome. Fertility Sterility (2018) 109(3):532–9. 10.1016/j.fertnstert.2017.11.020 29428305

[62] Heras V Castellano JM Fernandois D Velasco I Rodriguez-Vazquez E Roa J. Central Ceramide Signaling Mediates Obesity-Induced Precocious Puberty. Cell Metab (2020) 32(6):6–8. 10.1016/j.cmet.2020.10.001 33080217

[63] Schroeder B Backhed F. Signals From the Gut Microbiota to Distant Organs in Physiology and Disease. Nat Med (2016) 22(10):1079–89. 10.1038/nm.4185 27711063

[64] Cryan JF O’Riordan KJ Cowan CSM Sandhu KV Bastiaanssen TFS Boehme M. The Microbiota-Gut-Brain Axis. Physiol Rev (2019) 99(4):1877–2013. 10.1152/physrev.00018.2018 31460832

[65] Elkind-Hirsch K Marrioneaux O Bhushan M Vernor D Bhushan R. Comparison of Single and Combined Treatment With Exenatide and Metformin on Menstrual Cyclicity in Overweight Women With Polycystic Ovary Syndrome. J Clin Endocr Metab (2008) 93(7):2670–8. 10.1210/jc.2008-0115 18460557

[66] Nylander M Frossing S Clausen HV Kistorp C Faber J Skouby SO. Effects of Liraglutide on Ovarian Dysfunction in Polycystic Ovary Syndrome: A Randomized Clinical Trial. Reprod BioMed Online (2017) 35(1):121–7. 10.1016/j.rbmo.2017.03.023 28479118

[67] Salamun V Jensterle M Janez A Bokal EV. Liraglutide Increases Ivf Pregnancy Rates in Obese Pcos Women With Poor Response to First-Line Reproductive Treatments: A Pilot Randomized Study. Eur J Endocrinol (2018) 179(1):1–11. 10.1530/EJE-18-0175 29703793

[68] Outeirino-Iglesias V Romani-Perez M Gonzalez-Matias LC Vigo E Mallo F. Glp-1 Increases Preovulatory LH Source and the Number of Mature Follicles, As Well As Synchronizing the Onset of Puberty in Female Rats. Endocrinology (2015) 156(11):4226–37. 10.1210/en.2014-1978 26252058

[69] Heppner KM Baquero AF Bennett CM Lindsley SR Kirigiti MA Bennett B. Glp-1r Signaling Directly Activates Arcuate Nucleus Kisspeptin Action in Brain Slices But Does Not Rescue Luteinizing Hormone Inhibition in Ovariectomized Mice During Negative Energy Balance. eNeuro (2017) 4(1):ENEURO.0198-16.2016. 10.1523/ENEURO.0198-16.2016 PMC5247618 28144621

[70] Farkas I Vastagh C Farkas E Balint F Skrapits K Hrabovszky E. Glucagon-Like Peptide-1 Excites Firing and Increases Gabaergic Miniature Postsynaptic Currents (mPSCs) in Gonadotropin-Releasing Hormone (Gnrh) Neurons of the Male Mice *Via* Activation of Nitric Oxide (NO) and Suppression of Endocannabinoid Signaling Pathways. Front Cell Neurosci (2016) 10:214. 10.3389/fncel.2016.00214 27672360 PMC5018486

[71] Whelan K Efthymiou L Judd PA Preedy VR Taylor MA. Appetite During Consumption of Enteral Formula as a Sole Source of Nutrition: The Effect of Supplementing Pea-Fibre and Fructo-Oligosaccharides. Br J Nutr (2006) 96(2):350–6. 10.1079/BJN20061791 16923230

[72] Archer BJ Johnson SK Devereux HM Baxter AL. Effect of Fat Replacement by Inulin or Lupin-Kernel Fibre on Sausage Patty Acceptability, Post-Meal Perceptions of Satiety and Food Intake in Men. Br J Nutr (2004) 91(4):591–9. 10.1079/BJN20031088 15035686

[73] Samuel BS Shaito A Motoike T Rey FE Backhed F Manchester JK. Effects of the Gut Microbiota on Host Adiposity are Modulated by the Short-Chain Fatty-Acid Binding G Protein-Coupled Receptor, Gpr41. Proc Natl Acad Sci U S A (2008) 105(43):16767–72. 10.1073/pnas.0808567105 PMC2569967 18931303

[74] Tolhurst G Heffron H Lam YS Parker HE Habib AM Diakogiannaki E. Short-Chain Fatty Acids Stimulate Glucagon-Like Peptide-1 Secretion *Via* the G-protein-coupled Receptor Ffar2. Diabetes (2012) 61(2):364–71. 10.2337/db11-1019 PMC3266401 22190648

[75] Nohr MK Pedersen MH Gille A Egerod KL Engelstoft MS Husted AS. GPR41/FFAR3 and GPR43/FFAR2 as Cosensors for Short-Chain Fatty Acids in Enteroendocrine Cells vs FFAR3 in Enteric Neurons and FFAR2 in Enteric Leukocytes. Endocrinology (2013) 154(10):3552–64. 10.1210/en.2013-1142 23885020

[76] Hwang I Park YJ Kim YR Kim YN Ka S Lee HY. Alteration of Gut Microbiota by Vancomycin and Bacitracin Improves Insulin Resistance *Via* Glucagon-Like Peptide 1 in Diet-Induced Obesity. FASEB J (2015) 29(6):2397–411. 10.1096/fj.14-265983 25713030

[77] Strandwitz P. Neurotransmitter Modulation by the Gut Microbiota. Brain Res (2018) 1693(Pt B):128–33. 10.1016/j.brainres.2018.03.015 PMC6005194 29903615

[78] Hassan AM Mancano G Kashofer K Frohlich EE Matak A Mayerhofer R. High-Fat Diet Induces Depression-Like Behaviour in Mice Associated With Changes in Microbiome, Neuropeptide Y, and Brain Metabolome. Nutr Neurosci (2019) 22(12):877–93. 10.1080/1028415X.2018.1465713 29697017

[79] Liang Z Di N Li L Yang D. Gut Microbiota Alterations Reveal Potential Gut-Brain Axis Changes in Polycystic Ovary Syndrome. J Endocrinol Invest (2021). 10.1007/s40618-020-01481-5 33387350

[80] Dalile B Van Oudenhove L Vervliet B Verbeke K. The Role of Short-Chain Fatty Acids in Microbiota-Gut-Brain Communication. Nat Rev Gastroenterol Hepatol (2019) 16(8):461–78. 10.1038/s41575-019-0157-3 31123355

[81] Sampson TR Debelius JW Thron T Janssen S Shastri GG Ilhan ZE. Gut Microbiota Regulate Motor Deficits and Neuroinflammation in a Model of Parkinson’s Disease. Cell (2016) 167(6):1469–80 e12. 10.1016/j.cell.2016.11.018 27912057 PMC5718049

[82] Zhang JC Sun ZH Jiang SM Bai XY Ma CC Peng QN. Probiotic Bifidobacterium Lactis V9 Regulates the Secretion of Sex Hormones in Polycystic Ovary Syndrome Patients Through the Gut-Brain Axis. Msystems (2019) 4(2):e00017–19. 10.1128/mSystems.00017-19 31020040 PMC6469956

[83] Zhang BJ Shen SM Gu TW Hong T Liu JY Sun J. Increased Circulating Conjugated Primary Bile Acids are Associated With Hyperandrogenism in Women With Polycystic Ovary Syndrome. J Steroid Biochem (2019) 189:171–5. 10.1016/j.jsbmb.2019.03.005 30849463

[84] Jayasena CN Nijher GM Comninos AN Abbara A Januszewki A Vaal ML. The Effects of kisspeptin-10 on Reproductive Hormone Release Show Sexual Dimorphism in Humans. J Clin Endocrinol Metab (2011) 96(12):E1963–72. 10.1210/jc.2011-1408 PMC3232613 21976724

[85] Narayanaswamy S Jayasena CN Ng N Ratnasabapathy R Prague JK Papadopoulou D. Subcutaneous Infusion of Kisspeptin-54 Stimulates Gonadotrophin Release in Women and the Response Correlates With Basal Oestradiol Levels. Clin Endocrinol (Oxf) (2016) 84(6):939–45. 10.1111/cen.12977 PMC4914955 26572695

[86] Ely BR Francisco MA Halliwill JR Bryan SD Comrada LN Larson EA. Heat Therapy Reduces Sympathetic Activity and Improves Cardiovascular Risk Profile in Women Who are Obese With Polycystic Ovary Syndrome. Am J Physiol Regul Integr Comp Physiol (2019) 317(5):R630–40. 10.1152/ajpregu.00078.2019 PMC8424543 31483156

[87] Maliqueo M Benrick A Alvi A Johansson J Sun M Labrie F. Circulating Gonadotropins and Ovarian Adiponectin System are Modulated by Acupuncture Independently of Sex Steroid or Beta-Adrenergic Action in a Female Hyperandrogenic Rat Model of Polycystic Ovary Syndrome. Mol Cell Endocrinol (2015) 412:159–69. 10.1016/j.mce.2015.04.026 25963796

[88] Yoon HS Cho CH Yun MS Jang SJ You HJ Kim JH. Akkermansia Muciniphila Secretes a Glucagon-Like Peptide-1-Inducing Protein That Improves Glucose Homeostasis and Ameliorates Metabolic Disease in Mice. Nat Microbiol (2021) 6(5):563–73. 10.1038/s41564-021-00880-5 33820962

[89] Zhao L Zhang F Ding X Wu G Lam YY Wang X. Gut Bacteria Selectively Promoted by Dietary Fibers Alleviate Type 2 Diabetes. Science (2018) 359(6380):1151–6. 10.1126/science.aao5774 29590046

[90] Allegretti JR Kassam Z Mullish BH Chiang A Carrellas M Hurtado J. Effects of Fecal Microbiota Transplantation With Oral Capsules in Obese Patients. Clin Gastroenterol Hepatol (2020) 18(4):855–63.e2. 10.1016/j.cgh.2019.07.006 31301451

[91] Sun J Li H Jin Y Yu J Mao S Su KP. Probiotic Clostridium Butyricum Ameliorated Motor Deficits in a Mouse Model of Parkinson’s Disease *Via* Gut microbiota-GLP-1 Pathway. Brain Behav Immun (2021) 91:703–15. 10.1016/j.bbi.2020.10.014 33148438

[92] Olson CA Vuong HE Yano JM Liang QY Nusbaum DJ Hsiao EY. The Gut Microbiota Mediates the Anti-Seizure Effects of the Ketogenic Diet. Cell (2018) 173(7):1728–41.e13. 10.1016/j.cell.2018.04.027 29804833 PMC6003870

[93] Zheng P Zeng B Liu M Chen J Pan J Han Y. The Gut Microbiome From Patients With Schizophrenia Modulates the Glutamate-Glutamine-GABA Cycle and Schizophrenia-Relevant Behaviors in Mice. Sci Adv (2019) 5(2):eaau8317. 10.1126/sciadv.aau8317 30775438 PMC6365110

[94] Kootte RS Levin E Salojarvi J Smits LP Hartstra AV Udayappan SD. Improvement of Insulin Sensitivity After Lean Donor Feces in Metabolic Syndrome Is Driven by Baseline Intestinal Microbiota Composition. Cell Metab (2017) 26(4):611–9.e6. 10.1016/j.cmet.2017.09.008 28978426

[95] Chen L Wang D Garmaeva S Kurilshikov A Vich Vila A Gacesa R. The Long-Term Genetic Stability and Individual Specificity of the Human Gut Microbiome. Cell (2021) 184(9):2302–15.e12. 10.2139/ssrn.3653563 33838112

